# Parental depression and child conduct problems: evaluation of parental service use and associated costs after attending the Incredible Years Basic Parenting Programme

**DOI:** 10.1186/1472-6963-13-523

**Published:** 2013-12-18

**Authors:** Joanna M Charles, Tracey J Bywater, Rhiannon Tudor Edwards, Judy Hutchings, Lu Zou

**Affiliations:** 1 Centre for Health Economics and Medicines Evaluation (CHEME), Institute of Medical and Social Care Research, Bangor University, Dean Street Building, LL57 1UT Bangor, Gwynedd, UK; 2 Institute for Effective Education, University of York, Heslington, York, UK; 3 School of Psychology, Bangor University, Bangor, Gwynedd, UK; 4 Biostatistician at Barts and the London School of Medicine and Dentistry, Queen Mary University of London, London, UK

**Keywords:** Parenting, Depression, Service use, Health economics, Child conduct problems

## Abstract

**Background:**

There is co-morbidity between parental depression and childhood conduct disorder. The Incredible Years (IY) parenting programmes reduce both conduct disorder in children and depression in their parents. Recent U.K. and Ireland trials of the effectiveness and cost-effectiveness of IY parenting programmes have assessed children’s health and social care service use, but little is known about the programme’s impact on parental service use. This paper explores whether an above clinical cut-off score on the Beck Depression Inventory II (BDI II) is associated with high or low parental health and social care service use in high-risk families receiving the IY Basic Programme.

**Methods:**

This is a secondary analysis of a subsample (N = 119) from the first U.K. community-based randomised controlled trial of the 12-week IY Basic Programme (N = 153). Parents with children at risk of developing conduct disorder were randomised to receive the programme or to a waiting-list control group. BDI II total and BDI II clinical depression cut-off scores were compared to frequencies and costs of parents’ service use, at baseline, six, twelve and eighteen months post-baseline for the intervention group and at baseline and six months post-baseline for the control group.

**Results:**

Intervention group parents who scored above the clinical cut-off on the BDI II at baseline used more health and social care services than those who scored below at baseline, six and eighteen months. Significant reductions in service use frequencies were found for the intervention group only.

**Conclusion:**

Parents with higher levels or depression used more health and social care service and parenting programmes have been shown to reduce parental depression and also health and social service use. However, further exploration of depressed parents’ service use and the cost implications for publically funded health and social care services is needed.

**Trial registration:**

Registration of the original RCT of the IY Basic Parenting Programme - Current Controlled Trials ISRCTN46984318

## Background

Conduct disorder (CD) is defined as a persistent pattern of aggressive and destructive behaviours [1]. It is the most common psychiatric disorder in children [2]. Unipolar depression in adults, the most commonly occurring type of depression, is characterised by; enduring depressed mood, a loss of interest in previously enjoyed activities, disrupted sleep and/or appetite, feelings of guilt and/or low self-worth, and poor concentration [3]. Unipolar depressive disorder is expected to become the leading cause of global disease burden to services by 2015 [3].

There is a high rate of co-morbidity between depression in parents and CD in children [4-6]. There is also a link between low socio-economic status, depression, and conduct problems. Parents in socially-disadvantaged areas have higher prevalence of parental depression [7] and children in those areas have higher levels of CD [8].

Parenting interventions are the most effective method for improving negative or challenging child behaviour [9]. These interventions are typically delivered in a group format, one 2-hour session per week for 4–18 weeks, by trained leaders with the focus on improving parenting skills to manage child behaviour [10]. Effective parenting interventions tend to be multi-faceted, comprising of group discussion, role-play, video-modelling, and homework tasks and some have shown psychologically beneficial outcomes for parents (such as reductions in depression and stress levels) [11], and positive behavioural changes in children [12,13]. The following two sections present evidence for parenting programmes reducing parental depression. The third section presents associated costs of adult mental health issues and related child conduct problems.

### Parents attending a parent programme primarily for their improving child’s behaviour and their depression improved

Barlow, Coren and Stewart-Brown’s Cochrane review [14] identified four studies that assessed the impact of group based parenting programmes upon parental depression. DeGarmo, Patterson and Forgatch; Sheeber and Johnson; Scott and Stradling; Taylor et al. [15-18] all showed significant improvements in parental depression following participation in parenting programmes. The studies measured the effect of different parenting programmes delivered over a range of time-periods, using different depression sub-scales and child behaviour sub-scales to measure outcomes. Barlow, Coren and Stewart-Brown [14] concluded that, in the short-term, parenting programmes are effective in improving parental psychosocial outcomes. The DeGarmo, Patterson and Forgatch study [15] was the only one to assess longer-term outcomes, demonstrating that a change in child behaviour led to reductions in maternal depressive symptoms over 2.5 years. Four other more recent studies not included in the Barlow, Coren and Stewart-Brown review [14] also found improvements in parental depression as a secondary outcome from parenting programmes [5,12,19-21].

Furlong et al. [22] conducted a Cochrane review of behavioural and cognitive-behavioural group-based parenting programmes for early-onset conduct problems in children aged three to 12 years. Eight of the thirteen studies identified included self-reported parental mental health measures. Meta-analysis revealed a statistically significant small improvement in parent mental health, favouring the parent training group with confidence intervals indicating a range of small to moderate effect sizes. Hutchings et al. [23] have argued that since the reduction in depression co-occurs with the reduction in child behaviour problems for the intervention group only it is likely that the skills taught in the parent programme also contribute to the improvements in depression. They argue that the specific components that produce this change are training in accurate observation, behavioural rehearsal and problem solving skills all of which are known to be skill deficits in people experiencing depression.

### Mediator/moderator analyses conducted to demonstrate the effects of depression on child behaviour outcomes

The Hutchings et al. study [12] dataset has been used in mediator and moderator analyses. Findings showed maternal depression to be a significant positive moderator of child behaviour. Children whose mothers were depressed, as assessed by self-report on the Beck Depression Inventory II (BDI II) [24] showed greater improvements in conduct problems post-intervention, compared with parents of control group children [25]. This contrasts with other studies that have shown depression to be a significant moderator for poorer child behaviour outcomes using parenting programmes [26,27]. Hutchings et al. [23] using the same Hutchings et al. study [12] dataset found that parental depression partially mediated improvements in child behaviour. They argue that it is the collaborative nature of the IY programme, with its focus on empowering parents that makes it more effective in reducing both depression and child behavioural problems.

### Costs of child CD and adult depression

Previous research has shown the costs and frequencies of health, social and education service use of childhood CD and behavioural problems to be high [28,29]. There is research reporting the service use of children displaying problem behaviour [19-21,30]. In the Hutchings et al. sample [12], children’s service use was shown to increase at six and twelve months post-intervention, compared with service use at baseline [30]. However, long-term follow up of the same sample showed that children’s service use had decreased by eighteen months post-baseline [19]. Little investigation has been conducted to assess parental service use following participation in a parenting programme. Though Bywater et al. assessed the effects of foster carers’ service use in Wales [20]. Findings showed the majority of health and social care service use costs for the 42 foster carers in the study came from contacts with social workers with a total cost of £8,470 (mean cost of £202 (SD 207) per carer) over six months [20].

The costs of treating/alleviating depression in the NHS are high. Department of Health figures for the U.K. show that the total investment in adult mental health services is £5,892 billion a year (cost year 2008/09) [31]. Thomas and Morris estimated that the direct cost of treating depression, (predominately National Health Service costs) were around £370 million, including; in- and out-patient care, GP consultations and medication [32]. Previous research has also found individuals with high levels of depression use more services than those with lower levels of depression [33,34].

Based on previous findings this paper aims to;

1. Explore whether parents who score above the clinical cut-off on the BDI II [24] use more health and social services than parents who score below the cut-off.

2. Explore whether parental depression and frequencies of service use reduce following participation in a parent programme through secondary sub-sample analysis of a previously studied community sample of parents of children with conduct problems who participated in a 12-week IY parenting programme [12].

## Methods

This is a secondary analysis of data gathered in a randomised controlled trial (RCT) of the IY Basic Parenting Programme [12].

### Sample

**Figure 1:**
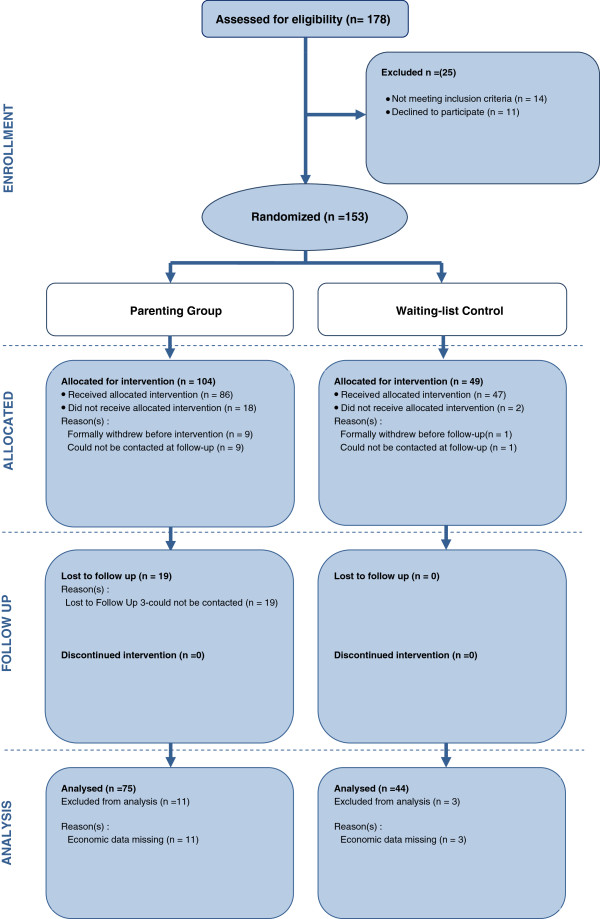
**WebCONSORT diagram of health economic sub-sample retention and participants lost to follow up for secondary analysis.** Legend: Flowchart of health economic sub-sample retention and participants lost to follow up for secondary analysis.

The original RCT sample consisted of 153 (Intervention N = 104, Control N = 49) parents of children aged 3–4 years old (at baseline) living in 11 disadvantaged Sure Start Areas in north and mid Wales who consented to take part (see [12,19,30] for further details of the original trial). Ethical approval for the original RCT was granted by the North West Wales research ethics committee (ref No 02/12) in accordance with the Helsinki Declaration. This was a targeted sample; parents were eligible to take part in the research if their child was ‘at risk’ of developing CD, as defined by scoring above the clinical cut off on either the Eyberg Child Behaviour Inventory (ECBI) Problem or Intensity Scale (11 or 127 respectively) [35]. Parents were randomly assigned, on a 2:1 ratio, to an intervention or a six-month waiting list control group. Randomisation was stratified by gender, and age of index child. The sample size for the sub-sample analysis used in this paper is smaller than the sample in the original trial. Complete service use and depression data was available for 119 parents (Intervention N = 75, Control N = 44) at baseline and six-month post-baseline follow-up, 75 intervention participants at twelve months and 56 intervention participants at eighteen months post-baseline (see Figure 1). Control families (N = 44) were offered the intervention after the six month follow up; therefore, are not included in the analyses past the six-month follow-up. Participants were all included irrespective of uptake of the intervention. Hutchings et al. reported an uptake rate of 9.2 mean sessions attended (SD 3.2) out of 12 sessions offered [12]. Intervention groups varied from 5 to 12 parents, with an average of seven parents attending the two-hour weekly sessions [12].

### Intervention

The IY Basic Parenting Programme is a 12-week group based programme (revised in 2008 to a 14–18 week programme) designed to equip parents (12-14/group) with the skills to manage challenging child behaviour through role-play, group discussion, video-modelling and homework activities [36]. The intervention was delivered at a cost of £1,934 per child (based upon eight children per group) and £1,289 (based upon 12 children per group. This cost includes initial costs and materials for training group leaders and related to the cost year 2003/04.

### Leaders

Sessions were facilitated by two group leaders who had attended the three-day basic leader training course. Group leaders were mostly Sure Start local children’s center staff, in some cases supported by Child and Adolescent Mental Health Services (CAMHS) primary care staff.

### Measures

The standardised and validated measures reported here are a sub-set of measures administered in the original trial [12]. The self-report parent measures of interest in the current evaluation are the BDI II [24] and the Client Service Receipt Inventory (CSRI) [37].

The BDI II [24] assesses depression with 21 items. The minimal depression range is 0–13, mild depression 14–19, moderate depression 20–28, and severe depression 29–63. The clinical cut off point for the BDI II is a score of 19 and over [24].

A modified version of the CSRI [37] was administered to assess parents’ and children’s contacts with health, social care and education professionals in the six months prior to completion of the measure.

In addition a Demographic Questionnaire, based on the Personal Development and Health Questionnaire (PDHQ) [38] was used to attain basic socio-demographic and general health data on family members.

### Procedure

Intervention group parents attended the 12-week IY Basic Parenting Programme between baseline and the six-month follow-up. Waiting-list control group parents were offered the intervention after the six-month follow-up interviews. Researchers conducted home visits to complete the measures at baseline and the six, twelve, and eighteen months post-baseline. Researchers were blind to allocation [12].

Analysis for this paper was performed for participants for whom complete data-sets of both the clinical and economic measures of interest were available across all time points. Prior to main analysis normality tests were undertaken, using Kolmogorov-Smirnov statistic and inspection of the Q-Q plots, and differences between demographic characteristics of the intervention and control groups at baseline. Four sets of analyses were conducted:

#### Assessing changes in depression scores over time

Participant depression scores, as measured by the BDI II [24], were compared over time for the intervention and control groups, using non-parametric Wilcoxon Signed Rank tests (using Bonferonni adjusted alpha in order to control for Type 1 errors).

#### Assessing changes in service use over time

Service use costs were calculated from a multi-agency public sector perspective using national costs for all time-points for the cost year 2009/10 [39,40]. Costs at eighteen months post-baseline were discounted at 3.5% in accordance with NICE guidelines [41]. Service use was divided into three categories; primary services consisting of GP, nurse and health visitor contacts, social services consisting of social worker, community psychiatric nurse, mediation service and counsellor contacts, and hospital services consisting of outpatient, inpatient and accident and emergency contacts. Costs of the IY Basic Parenting Programme were not included as a service use cost for the intervention group. Total service use frequencies and costs for each of the three categories within-participants were compared over time for both the intervention and control groups using non-parametric Friedman tests.

### Above or below clinical cut off score on the BDI II and frequencies of total service use

The clinical cut-off point was determined from the Beck et al. BDI II manual [24]. Non-parametric Mann–Whitney U tests were conducted to assess whether an above or below clinical cut off score on the BDI II [24] was associated with the frequency of total service use.

### Longitudinal analysis

A mixed between-within subjects ANOVA in SPSS version 16 was used to conduct longitudinal analysis for the intervention group to assess whether an above or below clinical cut off score on the BDI II [24] at baseline was associated with frequencies of total service use throughout the trial and subsequent follow ups. The independent between-subjects variable was the above or below clinical cut-off score on the BDI II [24]. The independent within-subjects variable was time, with four levels (baseline, 6, 12 and 18 month post-baseline scores) and one dependent variable, the total frequency of service use at each of the four time-points.

## Results

Table 1, below, describes the participant characteristics in the secondary sub-sample analysis. Sub-sample parents were not significantly different to parents in the main RCT sample. As in the main sample, there were more male than female children and the mean age of the children in months was the same [12]. No significant differences were found between the sub-sample intervention and control group at baseline (N = 119) using Mann–Whitney U tests prior to conducting analyses. Normality tests revealed non-significant differences at baseline using a Kolmogorov-Smirnov statistic for BDI II [24] and inspection of histograms and Q-Q plots supports these results.

**Table 1:** Family characteristics at all time-points for the secondary analysis

	***Baseline (N = 119)***				***6 months post-baseline (N = 119)***				***12 months post-baseline (N = 75)***		***18 months post-baseline (N = 56)***	
	***Intervention (n = 75)***		***Control (n = 44)***		***Intervention (n = 75)***		***Control (n = 44)***		***Intervention (n = 75)***		***Intervention (n = 56)***	
Parent sex: Males	1	(1.3%)	1	(2.3%)	1	(1.3%)	1	(2.3%)	1	(1.3%)	0	(0%)
Females	74	(98.7%)	43	(97.7%)	74	(98.7%)	43	(97.7%)	74	(98.7%)	56	(100%)
Child sex: Males	42	(56%)	30	(68.2%)	42	(56%)	30	(68.2%)	42	(56%)	31	(55.4%)
Females	33	(44%)	14	(31.8%)	33	(44%)	14	(31.8%)	33	(44%)	25	(44.6)
No of single mothers living alone	29	(38.7%)	14	(31.8%)	29	(38.7%)	14	(31.8%)	29	(38.7%)	23	(41.1%)
	*Mean*	*SD*	*Mean*	*SD*	*Mean*	*SD*	*Mean*	*SD*	*Mean*	*SD*	*Mean*	*SD*
Parents age (years)	29.4	(7.05)	28.0	(5.07)	29.4	(7.05)	28.0	(5.07)	29.4	(7.05)	29.2	(6.81)
Age of child (months) at baseline	46.2	(6.58)	46.2	(6.35)	46.2	(6.58)	46.2	(6.35)	46.2	(6.58)	46.09	(6.77)

Legend: Participating family characteristics at baseline, six, twelve, and eighteen months post-baseline for the secondary analysis sample.

Note: control group offered programme after six months and are therefore not included in 12 and 18-month analyses.

Normality tests using a Kolmogorov-Smirnov statistic revealed total frequency of service use at all four time-points, and BDI II [24] scores at six, twelve and eighteen months post-baseline were not normally distributed and inspection of histograms and Q-Q plots supported these results. Therefore, non-parametric tests have been used through the analysis; except in the case of the ANOVA, where a logarithmic transformation was performed on service use data using a small shift in the data to eliminate zero frequency counts. This method was used because non-parametric tests did not allow for comparisons of service use between participants who scored above clinical cut-off on the BDI II [24], and those intervention participants who scored below across all four time-points. Service use data was closer to a normal distribution after transformation; following inspection of the Q-Q plots and on the basis of central limit theorem as participant numbers exceeded 30 at each time point [42].

The results are described by the four types of analyses outlined in the methods.

### Changes in depression scores over time

**Figure 2:**
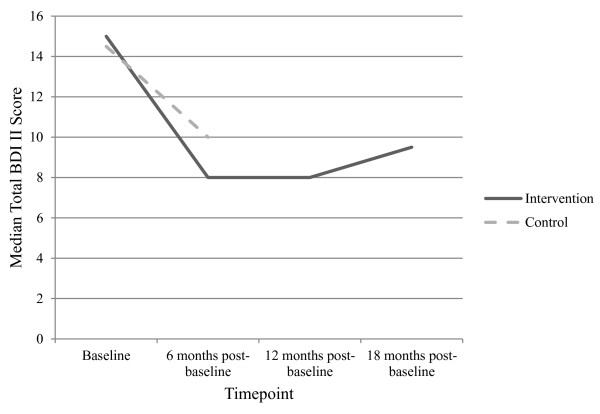
**Median parent BDI II**[24]**total scores for sub-sample families allocated to the intervention and control groups.** Legend: Median parent BDI II [24] raw and unadjusted total scores for sub-sample families allocated to the intervention and control group for the secondary analysis.

As expected this sub-sample reflected the main sample findings [19] (see Figure 2). Mann–Whitney U tests revealed no statistically significant difference between BDI II [24] total scores between the intervention group and control group at baseline and 6 months post-baseline.

Results of Friedman tests indicated a statistically significant difference for the intervention group in BDI II [24] total score across all four time-points, (baseline to eighteen months post-baseline) χ^2^ (3, N = 56) = 25.72, *p* < .05. No statistically significant difference was found in BDI II [24] total scores for the control group across the two time-points baseline to six months post-baseline (see Table 2 for Medians, Means and Standard Deviations of BDI II [24] total scores for both the intervention and control groups).

**Table 2:** **BDI II [**[24]**] total score for intervention and control groups**

***Time-point***	***BDI II total score intervention group***	***BDI II total score control group***
	***Median, Mean (SD)***	***Median, Mean (SD)***
Baseline	15, 16.80 (10.55)	14.50, 14.95 (9.62)
6 months post-baseline	8, 10.68 (9.98)	10.00, 13.25 (10.49)
12 months post-baseline	8, 10.83 (9.61)	---
18 months post-baseline	9.5, 12.36 (10.80)	---

Legend: BDI II [24] total score medians, means and standard deviations for intervention and control group participants at baseline, six, twelve, and eighteen months post-baseline for the secondary analysis sample.

Follow-up pair wise comparisons were conducted for the intervention group using Wilcoxon Signed Rank tests and controlling for the Type 1 errors across these comparisons at the 0.01 level (.05/7), a Bonferonni adjusted alpha value revealed a statistically significant reduction in BDI II [24] total score between baseline and six months post-baseline, z = −4.78, *p* < 0.01. A statistically significant reduction in BDI II [24] total score was found between baseline and twelve months post-baseline, z = −5.04, *p* < 0.01 and in BDI II [24] total score between baseline and eighteen months post-baseline, z = −3.56, *p* < 0.01.

### Changes in service use over time

**Figure 3:**
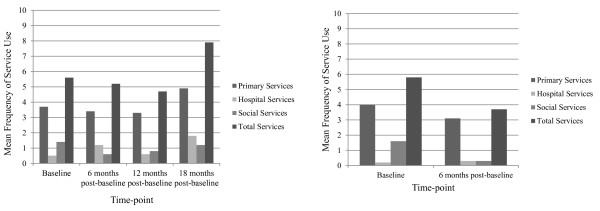
**Mean frequencies of health, social and hospital services used by parents in the sub-sample allocated to the intervention and control groups.** Legend: Mean frequencies of health, social and hospital services used by parents in the sub-sample, intervention and control groups as measured by the CSRI [37] which records service use in the preceding six months at the time of administration (n = 119).

**Figure 4:**
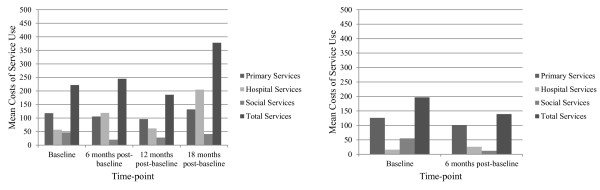
**Mean costs of health, social and hospital services used by parents in the sub-sample allocated to the intervention and control groups.** Legend: Mean costs of health, social and hospital services used by parents in the sub-sample, intervention and control groups as measured by the CSRI [37] which records service use in the preceding six months at the time of administration (n = 119)*†.* Costs were calculated from published national reference costs [39,40]. Costs were rounded up to the nearest pound, £. † Costs of service use between twelve and eighteen months post-baseline were discounted at 3.5%.

Figures 3 and 4 show the total mean frequencies as measured by CSRI [37], and associated costs of parental service use over 18 months.

Friedman tests indicated no statistically significant difference in total frequency of service use for the intervention group across the four time-points, baseline (Median = 3, Mean = 5.6, *S.D*. =7.42), six months post-baseline (Median = 2, Mean = 5.2, *S.D. =* 10.31), twelve months post-baseline (Median = 2, Mean = 4.7, *S.D*. = 7.49), and eighteen months post-baseline (Median = 3, Mean = 7.9, *S.D*. = 12.66). No statistically significant difference was found in total frequency of service use for the control group across the two time-points baseline (Median = 3.00, Mean = 5.8, S.D. = 6.65) to six months post-baseline (Median = 2.00, Mean = 3.7, *S.D*. = 5.56).

### Above or below clinical cut off score on the BDI II and frequencies of total service use

Tables 3 and 4 present mean frequencies and costs of service use for the sample depending upon whether parents scored above or below the clinical cut-off of 19 on the BDI II [24]. The number of parents scoring above clinical cut-off group decreased at six months for both intervention and control groups.

**Table 3:** **Mean frequencies and costs of service use split by BDI II [**[24]] cut-off for all parents assigned to the intervention group at all time-points^*†*±*^

	**Baseline (n = 75)**					**6 months post-baseline (n = 75)**					**12 months post baseline (n = 75)**					**18 months post-baseline† (n = 56)**				
	***Below cut-off (n = 46)***		***Above cut-off (n = 29)***			***Below cut-off (n = 62)***		***Above cut-off (n = 13)***			***Below cut-off (n = 59)***		***Above cut-off (n = 16)***			***Below cut-off (n = 42)***		***Above cut-off (n = 14)***		
CSRI service use categories	*Mean freq (s.d)*	*Mean cost £ (s.d)*	*Mean freq (s.d)*	*Mean cost £ (s.d)*	*P*	*Mean freq (s.d)*	*Mean cost £ (s.d)*	*Mean freq (s.d)*	*Mean cost £ (s.d)*	*P*	*Mean freq (s.d)*	*Mean cost £ (s.d)*	*Mean freq (s.d)*	*Mean cost £ (s.d)*	*P*	*Mean freq (s.d)*	*Mean cost £ (s.d)*	*Mean freq (s.d)*	*Mean cost £ (s.d)*	*P*
Primary services	2.2 (2.25)	64 (68.53)	6.2 (6.76)	202 (228.07)		2.2 (2.94)	66 (89.34)	8.9 (14.63)	298 (508.53)		3.7 (7.36)	108 (223.80)	1.7 (2.55)	53 (83.69)		4.1 (6.64)	105 (140.95)	7.4 (8.64)	215 (232.20)	
Hospital services	0.4 (1.90)	42 (104.41)	0.8 (1.29)	78 (121.24)		0.8 (1.80)	80 (164.44)	3.0 (7.65)	307 (720.80)		0.7 (1.58)	65 (146.97)	0.5 (1.75)	49 (184.84)		2.0 (7.41)	244 (879.06)	0.9 (1.56)	94 (164.29)	
Social services	0.5 (1.31)	17 (45.70)	2.8 (7.15)	94 (232.73)		1.2 (0.81)	9 (28.46)	2.3 (4.89)	72 (150.68)		1.0 (2.38)	34 (83.84)	0.3 (0.69)	8 (21.19)		0.5 (1.37)	15 (48.13)	3.6 (7.39)	120 (246.35)	
Total	3.1 (2.70)	123 (123.42)	9.8 (10.22)	374 (384.13)	*±*	4.2 (4.07)	155 (215.73)	14.2 (21.53)	677 (1130.64)	*±*	5.4 (8.17)	207 (294.11)	2.5 (3.47)	110 (200.93)		6.6 (12.64)	364 (950.19)	11.9 (12.33)	429 (388.68)	*±*

Legend: Parent mean total frequencies and costs of service use as measured by the CSRI [37] which records service use in the preceding six months at the time of administration for all families in the intervention group (n = 75) split by whether parent’s total BDI II [24] score was above or below the clinical cut-off *† ± .

* Costs were calculated from published national reference costs [39,40]. Costs were rounded up to the nearest pound, £.

† Costs of service use between twelve and eighteen months post-baseline were discounted at 3.5%.

*±* significant p = < .05.

**Table 4:** **Mean frequencies and costs of service use split by BDI II**[24]cut-off for all parents assigned to the control group at all time-point^*c*±*^

	**Baseline (n = 44)**					**6 months post-baseline (n = 44)**				
	***Below cut-off (n = 29)***		***Above cut-off (n = 15)***			***Below cut-off (n = 35)***		***Above cut-off (n = 9)***		
CSRI service use categories	*Mean freq (s.d)*	*Mean cost £ (s.d)*	*Mean freq (s.d)*	*Mean cost £ (s.d)*	*P*	*Mean freq (s.d)*	*Mean cost £ (s.d)*	*Mean freq (s.d)*	*Mean cost £ (s.d)*	*P*
Primary Services	2.8 (3.21)	91 (110.42)	6.2 (7.64)	194 (243.81)		3.2 (4.66)	105 (158.61)	2.7 (2.69)	87 (84.67)	
Hospital Services	0.1 (0.31)	10 (28.88)	0.3 (0.59)	28 (61.90)		0.2 (0.38)	16 (34.93)	0.7 (1.32)	63 (123.98)	
Social Services	0.8 (1.70)	24 (54.44)	3.1 (7.14)	113 (270.85)		0.3 (1.69)	11 (65.92)	0.4 (0.53)	17 (20.55)	
Total	3.7 (4.03)	125 (135.29)	9.6 (8.86)	335 (312.98)		3.7 (6.09)	132 (218.51)	3.8 (2.91)	167 (135.57)	

Legend: Parent mean total frequencies and costs of service use as measured by the CSRI [37] which records service use in the preceding six months at the time of administration for all families in the control group (n = 44) split by whether parent’s total BDI II [24] score was above or below the clinical cut-off * ± .

* Costs were calculated from published national reference costs c. Costs were rounded up to the nearest pound, £.

*±* significant p = < .05.

### Intervention group

Table 3 shows parents in the intervention group who scored above the BDI II [24] clinical cut-off had higher mean frequencies of total service use than parents who scored below the clinical cut –off across all four time-points. Mann–Whitney U tests revealed a statistically significant difference at baseline in the total frequency of services use by parents in the intervention group who scored below the BDI II [24] clinical cut-off and parents who scored above the BDI II [24] clinical cut-off *U* = 351, z = −3.47, *p* < .05. A statistically significant difference was found at six months post-baseline in the total frequencies of services use of parents in the intervention group who scored below the BDI II [24] clinical cut-off and those who scored above the clinical cut-off *U* = 250, z = −2.17, *p* < .05. A statistically significant difference was found at eighteen months post-baseline in the total frequency of services use by parents in the intervention group who scored below the BDI II [24] and parents who scored above and below the clinical cut-off *U* = 190, z = −1.99, *p* < .05.

No statistically significant differences were found at twelve months post-baseline in the total frequencies of services use for parents in the intervention group who scored below the BDI II [24] clinical cut-off and those who scored above the clinical cut-off.

### Control group

Table 4 shows parents in the control group who scored above the BDI II [24] clinical cut-off had higher mean frequencies of total service use than parents who scored below the clinical cut–off at baseline and at 6 months post-baseline. A Mann–Whitney U test revealed no statistically significant difference was found at baseline in the total frequencies of services use by parents in the control group who scored below the BDI II [24] clinical cut-off) and parents who scored above the clinical cut-off. No statistically significant difference was found at six months in the total frequencies of services use by parents in the control group who scored below the BDI II [24] clinical cut-off and parents who scored above the clinical cut-off.

### Longitudinal analysis

A mixed between-within subjects ANOVA showed a statistically significant effect for BDI II [24] clinical cut off, *F* (1, 54) = 9.99, *p* < .05. Parents in the intervention group who scored above the BDI II [24] clinical cut-off score at baseline, had increased service use throughout the trial, when compared with parents who scored below the clinical cut-off.

## Discussion

Parental depression decreased at six months for both the intervention and control groups; however, this decrease was only significant for the intervention group. BDI II [24] clinical cut-off was shown to impact upon service use; with parents who scored above the clinical cut-off utilising more services than parents who scored below the cut-off; findings are discussed in more detail below.

### Changes in depression over time

Total mean BDI II [24] depression scores decreased for both the intervention and control groups in the sub-sample. The differences were significant for the intervention group, but not for the control group. The larger, significant reduction in BDI II [24] mean total scores found in the intervention group at six months post-baseline could be attributed to the skills that were trained in the intervention including observation, realistic goal setting and problem solving, deficits in all of which are deficits associated with depression [23]. Improved parent/child relationships and reductions in negative child behaviour have been found post-intervention in previous RCTs of the IY parenting series [12,13,19,22]. In contrast to the main trial papers [12,19] no significant difference was found between the intervention and control group baseline to six months post-baseline. Although they did not differ at baseline to the intervention group, and the study was powered to look at these diffs at 2:1. This could be attributed to the smaller sample size used for the secondary sub-analysis.

### Changes in service use over time

Parents accessed a high number of services, with primary health services accounting for the highest frequencies and costs.

Total mean costs of service use for the intervention group increased at six and eighteen months post-baseline; however, costs decreased at twelve months post-baseline. These findings are not reflected in the decreases in mean total frequencies of service use. This could be attributed to participants using higher frequencies of more costly health and social care services such as hospital outpatient procedures, the study retained more of the high service users than the low service users at 18 months post-baseline. Costs of service use for the control group did not decrease significantly from baseline to six months post-baseline. Though this could again be attributed to the smaller sample size used for the secondary sub-analysis.

### BDI II clinical cut-off and service use

Findings demonstrate that a clinical level of self-reported parental depression affects the frequency and cost of health and social service use for parents in both intervention and control groups. Parents who scored above the clinical cut-off on the BDI II [24] in the intervention group accessed more health and social services than those who scored below at baseline, six and eighteen months post-baseline. Parents in the control group who scored above the clinical cut-off on the BDI II [24] also had higher mean frequencies and costs of service use than those who scored below the clinical cut off at baseline and six months post-baseline. However, these differences were not significant for the control group, though this may be attributed to the small sample size of the control group, which was less than half the size of the intervention group. The size of the control group was further reduced following delineation by BDI II [24] clinical cut-off, which is a further limitation of post-hoc sub-sample analyses in general.

These findings support previous data reporting that high levels of adult depression lead to high service use [33,34]. A mixed between-within subjects ANOVA revealed a significant effect for an above BDI II [24] clinical cut-off score at baseline, with increased service use throughout the trial for the intervention group, when compared with parents who scored below the clinical cut-off and for whom data were available at six months post baseline. These findings suggest that reducing clinical levels of depression as early as possible could result in decreased service use.

### Wider benefits of parenting programmes

Often, when evaluating interventions the wider impacts such as familial benefits are unexplored by researchers. In previous studies of parenting programmes, the literature has presented outcomes directly linked to children (e.g., child behaviour and/or children’s service use) [5,12,19,21,30]. Relatively little attention has been given to other potential benefactors of parenting programmes such as parents, siblings and extended family. Many public health interventions have more than one primary outcome; some may even have a range of equally important outcomes. Health economists should explore multiple intervention outcomes; unusually in the case of IY studies, UK and Irish health economists have explored child behaviour, parental depression and the use of public sector health and social care services [21,30]. Weatherly et al. [43] advocate using a cost-benefit approach for economic evaluations of public health interventions, such as parenting programmes, in order to consider all costs and benefits. This would enable researchers to assign a primary outcome measure such as child behaviour or service use, but also assign secondary outcomes, such as parental depression, parental service use, sibling behaviour or sibling service use. Hutchings et al. and Bywater et al. [12,19] explored the impact of the IY Basic parenting programme on siblings of the referred child. Intervention group parents reported significantly less severe intensity of problems in siblings measured by the ECBI [35] at follow-up, compared with siblings in the control group. Although clinical outcomes were measured for siblings, no assessment of sibling service use benefit was undertaken.

### Limitations and future research

The study was limited by a smaller sample size than available in the main RCT. This was due to lack of both complete clinical and economic data which was required for the analysis. The control group was less than half the size of the intervention group, due to 2:1 randomisation in the main RCT; however, it should be noted the main RCT was powered sufficiently. Comparisons could not be made between intervention and control groups long-term as the control families were offered the intervention after the six-month follow-up. Though it is unethical to deny families an effective intervention, the wait-list control condition presents a limitation of lack of long-term control group data, which would be useful to conduct long-term comparisons 12, 18 months post-baseline. This is a limitation across the field; the Barlow, Coren and Stewart-Brown and Furlong et al. reviews both concluded that, in the short-term, parenting programmes are effective in improving parental psychosocial outcomes and parental mental health, but neither review found many long-term studies with a comparator control group [14,22]. One long-term study identified in Barlow, Coren and Stewart-Brown’s (2009) review, found that reductions in children’s behaviour problems led to reductions in mothers’ depressive symptoms over 2.5 years [15]. Bywater et al. and Hutchings, Lane and Kelly [19,44] found maintained improvements in parental depression over four years for intervention versus standard treatment families. However, the general lack of long-term studies needs to be addressed in order to build effective interventions that continue to prove beneficial to families as the child grows.

## Conclusions

Parents are beneficiaries of parenting programmes. The skills that parents learn at the programme help them to interact more effectively with their children, and provide additional benefits such as reduced depression and stress [5,12,19,23,44]. Evaluations of the IY programmes have demonstrated reduced parental depression and are effective and cost-effective in improving child behaviour and decreasing the frequency of child service use [12,19,30].

To our knowledge this paper is the first to explore the service cost implications that a parent programme can have on the health and social care service use of biological parents. Results demonstrate that parents who scored above clinical cut-off on the BDI II [24] accessed more health and social services than those who scored below the cut-off. Thus reducing clinical levels of depression as early as possible could result in decreased service use, which could in turn reduce the financial burden upon publically funded national health and social care services. This paper also highlights the need for further exploration of the wider benefits of the IY parenting programme to benefactors other than the target child.

## Key messages

● A co-morbid link exists between parental depression and child behavioural problems. The Incredible Years Basic parent program reduces child behaviour problems and self-reported parental depression.

● There is a paucity of research on parents’ costs of health and social service use, following participation in a parenting program. Previous research has explored the impact of child conduct disorder upon the costs of publicly resourced services such as health and social care.

● Clinical levels of depression were associated with increased use and costs of health and social care service use, which can be reduced following attendance at a parenting program.

● To gauge the overall effectiveness of parenting programs, wider family health, behavioural and costs benefits (e.g. outcomes for siblings) need to be investigated as part of the assessment of impact.

● This paper illustrates the methodological challenge of undertaking economic evaluations of public health interventions with more than one main trial outcome.

## Competing interests

The authors declare that they have no competing interests.

## Authors’ contributions

JMC conducted the secondary analysis and produced a first draft of the manuscript. LZ provided statistical guidance to JMC. JH was principle investigator of the original evaluation of the IY Basic Parenting Programme, with TB and RTE as co investigators. TB and RTE co-supervised JMC during her PhD studentship. All authors read and commented on drafts and approved the final manuscript.

## Pre-publication history

The pre-publication history for this paper can be accessed here:

http://www.biomedcentral.com/1472-6963/13/523/prepub

## References

[B1] American Psychiatric Association Diagnostic and Statistical Manual of Mental Disorders, Fourth Edition Text Revision (DSM-IVTR) 2000 Washington, DC, USA: American Psychiatric Association

[B2] Meltzer H Gatward R Goodman R Ford T The mental health of children and adolescents in Great Britain http://www.dawba.info/abstracts/B-CAMHS99_original_survey_report.pdf 10.1080/0954026021000046155 12745331

[B3] World Health Organisation (WHO) The global burden of disease: 2004 update http://www.who.int/healthinfo/global_burden_disease/GBD_report_2004update_full.pdf

[B4] Alpern L Lyons-Ruth K Preschool children at social risk: chronicity and timing of maternal depressive symptoms and child behaviour problems at school and at home Dev Psychopathol 1993 13 371 387 10.1017/S0954579400004478

[B5] Hutchings J Appleton P Smith M Lane E Nash S Evaluation of two treatments for children with severe behaviour problems: child behaviour and maternal mental health outcomes Behav Cogn Psychother 2002 13 279 295

[B6] Lahey BB Piacentini JC McBurnett K Stone P Hartdaghn S Hynd G Psychopathology in the parents of children with conduct disorder and hyperactivity J Am Acad Child Adolesc Psychiatry 1988 13 163 170 10.1097/00004583-198803000-00005 3360717

[B7] Farrington DP The development of offending and antisocial behaviour from childhood: key findings from the Cambridge Study in Delinquent Development J Child Psychol Psychiatry 1995 13 929 964 10.1111/j.1469-7610.1995.tb01342.x 7593403

[B8] Attride-Stirling J Davis H Day C Selare I Someone to talk to who’ll listen: addressing the psychosocial needs of children and families J Community Appl Soc Psychol 2001 13 179 191 10.1002/casp.613

[B9] National Institute for Health and Clinical Excellence Parent-training/education programmes in the management of children with conduct disorders http://www.nice.org.uk/nicemedia/pdf/TA102guidance.pdf

[B10] Hutchings J Lane E The role of parenting to the development and prevention of child mental health problems Curr Opin Psychiatry 2005 13 386 391 10.1097/01.yco.0000172056.63401.e0 16639130

[B11] Mihalic S The importance of implementation fidelity Rep Emot Behav Disord Youth 2004 13 83 105

[B12] Hutchings J Bywater T Daley D Gardner F Whitaker C Jones K Eames C Edwards RT Parenting intervention in sure start services for children at risk of developing conduct disorder: pragmatic randomised controlled trial BMJ 2007 13 678 681 10.1136/bmj.39126.620799.55 17350966 PMC1839187

[B13] Webster-Stratton C Hancock L Schaefer CE Parent training for young children with conduct problems. Content, methods and therapeutic process Handbook of parent training 1998 New York: Wiley 98 152

[B14] Barlow J Coren E Stewart-Brown S Parent-training programmes for improving maternal psychosocial health Cochrane Database Syst Rev 2003 4 CD002020 DOI: 10.1002/14651858.CD002020.pub2 10.1002/14651858.CD002020 11034741

[B15] DeGarmo DS Patterson GR Forgatch MS How do outcomes in a specified parent training intervention maintain or wane over time? Prev Sci 2004 13 73 89 15134313 10.1023/b:prev.0000023078.30191.e0

[B16] Sheeber LB Johnson JH Evaluation of a temperament-focused, parent-training programme J Clin Child Psychol 1994 13 249 259 10.1207/s15374424jccp2303_3

[B17] Scott MJ Stradling SG Evaluation of a group programme for parents of problem children Behav Psychother 1987 13 224 239 10.1017/S0141347300012313

[B18] Taylor TK Schmidt F Pepler D Hodgins CA A comparison of eclectic treatment with Webster-Stratton’s parents and children series in a children’s mental health center: a randomised controlled trial Behav Ther 1998 13 221 240 10.1016/S0005-7894(98)80004-X

[B19] Bywater T Hutchings J Daley D Whitaker C Yeo ST Jones K Eames C Tudor Edwards R Long-term effectiveness of a parenting intervention in Sure Start services in Wales for children at risk of developing conduct disorder Br J Psychiatry 2009 13 318 324 10.1192/bjp.bp.108.056531 19794200

[B20] Bywater T Hutchings J Linck P Whitaker C Daley D Yeo ST Edwards RT Incredible Years parent training support for foster carers in Wales: a multi-center feasibility study Child Care Health Dev 2010 13 233 243 20854449 10.1111/j.1365-2214.2010.01155.x

[B21] O’Neill D McGilloway S Donnelly M Bywater T Kelly P A cost-effectiveness analysis of the Incredible Years parenting programme in reducing childhood health inequalities Eur J Health Econ 2013 13 85 94 10.1007/s10198-011-0342-y 21853340

[B22] Furlong M McGilloway S Bywater T Hutchings J Smith SM Donnelly M Behavioural and cognitive-behavioural group-based parenting programmes for early-onset conduct problems in children aged 3 to 12 years (Review) Cochrane Database Syst Rev 2012 2 CD008225 DOI: 10.1002/14651858.CD008225.pub2 22336837 10.1002/14651858.CD008225.pub2 PMC12935172

[B23] Hutchings J Bywater T Williams ME Lane E Whitaker C Improvements in parental depression as a mediator of child behaviour change Psychology 2012 13 795 801 10.4236/psych.2012.329120

[B24] Beck AT Steer RA Brown GK Manual for the Beck Depression Inventory-II 1996 San Antonio, Texas: Psychological Corporation

[B25] Gardner F Bywater JT Hutchings T Whitaker C Who benefits and how does it work? Moderators and mediators of outcomes in a randomised trial of parenting interventions in multiple ‘Sure Start’ services J Clin Child Psychol 2010 13 568 580 10.1080/15374416.2010.486315 20589567

[B26] Griest DL Forehand R Wells KC Follow-up assessment of parent behavioural training: an analysis of who will participate Child Study Journal 1981 13 221 229

[B27] Webster-Stratton C Hammond M Predictors of treatment outcome in parent training for families with conduct problem children Behav Ther 1990 13 319 337 10.1016/S0005-7894(05)80334-X

[B28] Knapp M Scott S Davies J The cost of antisocial behaviour in younger children a pilot study of economics and family impact J Clin Child Psychol Psychiat 1999 13 457 473 10.1177/1359104599004004003

[B29] Romeo R Knapp M Scott S Economic cost of severe anti-social behaviour in children - and who pays it Br J Psychiatry 2006 13 547 553 10.1192/bjp.bp.104.007625 16738345

[B30] Edwards RT Ó Céilleachair AJ Bywater T Hutchings J A parenting programme for children at risk of developing conduct disorder: a cost-effectiveness analysis BMJ 2007 13 334 682 10.1136/bmj.39126.699421.55 PMC1839236 17350965

[B31] Department of Health The 2008/09 National Survey of Investment in Mental Health Services http://www.dh.gov.uk/prod_consum_dh/groups/dh_digitalassets/documents/digitalasset/dh_103198.pdf

[B32] Thomas CM Morris S Cost of depression among adults in England in 2000 Br J Psychiatry 2003 13 514 519 10.1192/bjp.183.6.514 14645022

[B33] Herrman H Patrick DL Diehr P Martin ML Fleck M Simon GE Buesching DP and The LIDO Group Longitudinal investigation of depression outcomes in primary care in six countries: the LIDO study. Functional status, health service use and treatment of people with depressive symptoms Psychol Med 2002 13 889 902 12171383 10.1017/s003329170200586x

[B34] Johnson J Weissman MM Klerman GL Service utilization and social morbidity associated with depressive symptoms in the community JAMA 1992 13 1478 1483 10.1001/jama.1992.03480110054033 1538538

[B35] Eyberg SM Eyberg child behaviour inventory J Clin Child Psychol 1980 13 27

[B36] Webster-Stratton C Randomised trial of two parent-training programmes for families with conduct-disordered children J Consult Clin Psychol 1984 13 666 678 6470293 10.1037//0022-006x.52.4.666

[B37] Beecham J Knapp M Thomicroft G, Brewin C, Wing J Costing psychiatric interventions Measuring mental health needs 1992 1 London: Gaskell 163 183

[B38] Hutchings J The personal and parental characteristics of preschool children referred to a child and family mental health service and their relation to treatment outcome 1996 Bangor: PhD thesis. University of Wales

[B39] Curtis L Unit Costs of Health and Social Care 2009 Canterbury: Personal Social Services Research Unit, University of Kent

[B40] Department of Health National Health Service (NHS) reference costs 2008–2009 http://www.dh.gov.uk/en/Publicationsandstatistics/Publications/PublicationsPolicyAndGuidance/DH_111591

[B41] National Institute for Health and Clinical Excellence Guide to the Methods of Technology Appraisal http://www.nice.org.uk/media/B52/A7/TAMethodsGuideUpdatedJune2008.pdf 27905712

[B42] Field AP Discovering statistics using SPSS 2009 3 London: Sage Publications LTD

[B43] Weatherly H Drummond M Claxton K Cookson R Ferguson B Godfrey C Rice N Sculpher M Sowden A Methods for assessing the cost-effectiveness of public health interventions: key challenges and recommendations Health Policy 2009 13 85 92 10.1016/j.healthpol.2009.07.012 19709773

[B44] Hutchings J Lane E Kelly J Comparison of two treatments of children with severely disruptive behaviours: a four year follow up Behav Cogn Psychother 2004 13 15 30 10.1017/S1352465804001018

